# *In utero* and early-life exposure to thirdhand smoke causes profound changes to the immune system

**DOI:** 10.1042/CS20201498

**Published:** 2021-04-23

**Authors:** Antoine M. Snijders, Mi Zhou, Todd P. Whitehead, Briana Fitch, Priyatama Pandey, Aaron Hechmer, Abel Huang, Suzaynn F. Schick, Adam J. de Smith, Adam B. Olshen, Catherine Metayer, Jian-Hua Mao, Joseph L. Wiemels, Scott C. Kogan

**Affiliations:** 1 Biological Systems and Engineering Division, Lawrence Berkeley National Laboratory, Berkeley, CA, U.S.A.; 2 Department of Laboratory Medicine, University of California, San Francisco, CA, U.S.A.; 3 School of Public Health, University of California, Berkeley, CA, U.S.A.; 4 Center for Genetic Epidemiology, Keck School of Medicine, University of Southern California, Los Angeles, CA, U.S.A.; 5 UCSF Helen Diller Family Comprehensive Cancer Center, University of California, San Francisco, CA, U.S.A.; 6 Division of Occupational and Environmental Medicine, Department of Medicine, University of California, San Francisco, CA, U.S.A.; 7 Department of Epidemiology and Biostatistics, University of California, San Francisco, CA, U.S.A.

**Keywords:** immunology, leukemia, lymphoma, thirdhand smoke

## Abstract

Acute lymphoblastic leukemia (ALL) is the most common cancer in children. Thirdhand smoke (THS) is the residual tobacco contamination that remains after the smoke clears. We investigated the effects of THS exposure *in utero* and during early life in a transgenic *Cdkn2a* knockout mouse model that is vulnerable to the development of leukemia/lymphoma. Female mice, and their offspring, were exposed from the first day of pregnancy to weaning. Plasma cytokines, body weight and hematologic parameters were measured in the offspring. To investigate THS exposure effects on the development of leukemia/lymphoma, bone marrow (BM) was collected from control and THS-exposed mice and transplanted into BM-ablated recipient mice, which were followed for tumor development for 1 year. We found that *in utero* and early-life THS exposure caused significant changes in plasma cytokine concentrations and in immune cell populations; changes appeared more pronounced in male mice. Spleen (SP) and BM B-cell populations were significantly lower in THS-exposed mice. We furthermore observed that THS exposure increased the leukemia/lymphoma-free survival in BM transplantation recipient mice, potentially caused by THS-induced B-cell toxicity. A trend towards increased solid tumors in irradiated mice reconstituted with THS-exposed BM stimulates the hypothesis that the immunosuppressive effects of *in utero* and early-life THS exposure might contribute to carcinogenesis by lowering the host defense to other toxic exposures. Our study adds to expanding evidence that THS exposure alters the immune system and that *in utero* and early-life developmental periods represent vulnerable windows of susceptibility for these effects.

## Introduction

Acute lymphoblastic leukemia (ALL) is the most common type of childhood malignancy, with more than 50000 children diagnosed worldwide yearly, and 80% of these leukemias are B lymphoblastic. In the U.S., this disease has been increasing ∼1% per year for decades [1]. Although survival with childhood ALL has improved considerably in the last two decades due to more effective treatments, survivors face a lifelong battle with various medical conditions (e.g. hormonal, cardiovascular and pulmonary abnormalities, osteoporosis, secondary cancer) and neuropsychological problems (e.g. neurocognitive impairment, anxiety, depression, post-traumatic distress) as a result of treatments [2,3]. Thus, deciphering the etiology of pediatric leukemia remains an important goal.

The development of childhood ALL involves genetic and epigenetic processes, but the connection between specific environmental exposures and acquired tumor genetic and epigenetic changes in leukemia cells is inherently difficult to study in human populations. Incidence of childhood leukemia has steadily increased in the last half century, particularly among Latinos [1,4]. This increase is mainly accounted for by one leukemia subtype—common CD10^+^, CD19^+^ childhood pre-B cell ALL. The causes for this increase have been hypothesized to include exposures to chemicals (e.g. tobacco smoke, pesticides), dietary factors, fetal growth rates and patterns of infection [5,6]. Here, for the first time, we assessed the effect of exposure to ‘thirdhand smoke’ (THS), the residue left on surfaces after smoking, on the development of ALL.

Approximately 1.1 billion people are current smokers worldwide and this figure is expected to rise to over 1.6 billion by the year 2025 [7]. In many places, smoke-free laws ban smoking in public places; however, many children continue to be exposed to environmental tobacco smoke at home. Most studies on health effects of tobacco exposure in young children concentrate on passive smoking such as ‘secondhand smoke’ (SHS), which is the aerosol present while smoking is taking place. Exposure to SHS by non-smokers, primarily through inhalation, affects immune cell numbers, levels of cytokines and is associated with an increased risk of respiratory tract infections and cancer [8]. In recent years, attention has been brought to potential adverse health effects of pollutants that remain on surfaces and in dust after tobacco has been smoked (referred to as THS). These pollutants can be re-emitted into the gas-phase, or react with other compounds in the environment to form secondary pollutants [9–11]. Evidence supports the widespread presence of THS in indoor environments, including in the U.S. [12,13]. THS poses a potential health risk for children who tend to spend more time indoors than adults and have age-specific behaviors that bring them in closer contact with surfaces and dust. Moreover, their higher respiration rate relative to body size, larger exposed surface area to volume ratio, thinner skin, less mature immunologic systems and lower metabolic capacity could lead to increased THS exposure levels in children compared with adults. Similar to SHS, THS-exposed mice showed alterations in cytokine levels and immune cell numbers [14–16]. These prior studies focused on early-life exposure windows leaving the effects of THS exposure during the perinatal period unknown.

Abnormalities in *Cdkn2a* are observed in approximately one-third of childhood ALL [17]. In this study, we used our established *Cdkn2a* mouse model of childhood ALL [18] to investigate THS exposure effects during pregnancy and early life on the immune system of these leukemia-predisposed animals.

## Materials and methods

### Mice exposed to THS

All animal experimental protocols were approved by the University of California at San Francisco, Institutional Animal Care and Use Committee (UCSF-IACUC). The animal experiments were all performed in a specific pathogen-free facility at the University of California, San Francisco (UCSF) and carried out in accordance with the Guide for the Care and Use of Laboratory Animals of the National Institutes of Health. B-cell lymphoma development was previously described in *Cdkn2a* null mice [19], including the mice used in the present study [18]. In brief, our mice were derived from FVB/N *Cdkn2*^atm2Brn^ mice (MGI: 2384163) containing a floxed allele of *Cdkn2a* crossed with FVB/n-Tg(EIIa-cre)^C5379mgd/J^ mice (MGI:2137691) to generate *Cdkn2a* null animals on a pure FVB/N background. Terrycloth substrates were used as surrogates for indoor surfaces, on to which fresh SHS gases could adsorb and SHS particles deposit as previously described [20]. Briefly, clean 100% cotton terrycloth samples were repeatedly exposed to SHS in a 6-m^3^ stainless steel chamber for a total of 234 h over 1019 days. A total of 2795 mg of total particulate material was introduced into the steel chamber, which is equivalent to the smoke from 200 to 350 cigarettes over 2 years and 9 months. If all THS mass deposited on the surfaces of the exposure chamber, the maximum loading of THS on each gram of cotton cloth would be 238 μg. The THS cloth was removed from the smoke, vacuum-packed in Mylar film and stored at −20°C until use. THS compounds in terrycloth substrates were analyzed following the procedures described in our previous study and the same batch of cloth was used in this study [20]. Briefly, 0.85 g THS-laden and unexposed (control) cotton cloth samples were immersed in 10 ml Dulbecco’s Modified Eagle’s Medium (DMEM). Twelve targeted THS compounds were analyzed using liquid chromatography-tandem mass spectrometry (LC-MS/MS). Nicotine was detected at 30600 ng/g in THS cloth compared with 14.9 ng/g in control cloth. Other THS constituents detected in THS cloth, but not control cloth included myosamine (2440 ng/g), N-formylnornicotine (998 ng/g) and cotinine (486 ng/g) were detected in THS cloth, but not in control cloth. The levels of polycyclic aromatic hydrocarbons (PAH) were measured by gas chromatography coupled with mass spectrometry (GC/MS, Varian 4000, CA) after 2.5 × 5 cm specimens of the THS-laden and unexposed (control) cotton cloth samples were extracted with dichloromethane (DCM). Naphthalene (27 ng/g), 2-methyl naphthalene (27 ng/g) and pyrene (24 ng/g) were most abundant among the 12 detected PAHs in THS-laden cloth samples. PAH levels in control cloth were all below the level of quantitation.

The pregnant female *Cdkn2a*^−/−^ mice (FVB/N strain) were divided into two groups: control group (30 mice) and experimental group (32 mice), one female mouse per cage. The experimental group was exposed to one THS-exposed terrycloth swatch (5 × 5 cm^2^) and the control group was exposed to one sham cloth (5 × 5 cm^2^) from the first day of pregnancy till the pups were weaned. THS-exposed cloth or sham cloth was added to the standard bedding in the cages and was replaced once a week. Body weight of pups was measured at age of 3 weeks.

### Measurement of cytokine levels in mouse plasma samples

One male and one female two-day-old pups per independent litter were selected and killed by CO_2_ for 5 min followed by decapitation. A total of 60–70 µl of blood was collected in a K_2_EDTA pediatric blood vial from the selected pup. Blood samples were centrifuged at 14000 rpm for 5 min to collect the supernatant plasma sample (∼30 µl/sample) and saved in 1.5 ml DNase/RNase-free Eppendorf tubes at −80°C prior to analysis. The Luminex assay of cytokines (Supplementary Table S1) was performed following the protocol of the cytokine assay kits (Bio-plex Pro™ Mouse Cytokine standard 23-Plex, Group I, Cat. M600009RDPD; Bio-plex Pro™ Mouse Cytokine standard 9-Plex, Group II, Cat. MD000000EL) purchased from Bio-Rad Laboratory (Hercules, CA). Every step was performed as described in the Bio-Rad protocol except for a reduction in reagent volumes (10 µl of bead mixture, 10 µl of detection Antibody cocktail) and lower sample volumes (10 µl of four-fold diluted sample plasma) with the help of the Curiox DropArray wall-less microplate and Curiox plate washer (Curiox Biosystem, San Carlos, CA). The developed samples were suspended in 55 µl of sheath fluid and the fluorescence intensity (FI) of each sample was acquired by Bio-plex 200 plate reader system (Bio-Rad Laboratories, Inc.). The mean values of FI were calculated by comparing with the standard curve of each cytokine to determine the cytokine levels in each sample.

### Bone marrow flow cytometry and transplantation

When pups were 5 weeks old, control and THS-exposed mice (details noted in Supplementary Table S3) were selected as the bone marrow (BM) transplantation donors. Following inhalant isoflurane anesthesia, peripheral blood (PB) samples were collected by submandibular bleeding into EDTA-coated tubes (Becton Dickinson and Company, NJ) and the complete blood cell count (CBC) including red blood cell (RBC), white blood cell (WBC), neutrophil (NE), lymphocyte (LY), monocyte (MO) and platelet (PLT) was acquired by Hematology Analyzer (HemaVet950FS, Drew Scientific, Miami Lake, FL). Live non-erythroid cells were isolated from the PB, BM and spleen (SP) from 20 donor mice (10 mice from each group) following standard laboratory protocols. Subsequently, one million of those cells were incubated with fluorescent conjugated antibodies detecting subpopulations of B cells [B220^+^/CD19^+^; mature B cells (B220^+^/CD19^+^/IgK^+^) and immature B cells (B220^+^/CD19^+^/IgK^−^)], T cells (CD3^+^/CD4^+^ or CD3^+^/CD8^+^) and myeloid cells [CD19^−^/CD11b^+^; monocytes (CD19^−^/CD11b^+^/Gr-1^neg-lo^) and neutrophils (CD19^−^/CD11b^+^/Gr-1^mod-hi^)]. Antibody details are provided in Supplementary Table S4. The cells were analyzed on a SP6800 spectral analyzer (Sony Biotechnology Inc.) and the percentages of cell populations were delineated with FlowJo software.

A quantity of 2 × 10^6^ cells isolated from BM of each donor mouse were transplanted by retro-orbital injection into one female recipient FVB/N CD45.2 congenic animal after irradiation treatment (9.5 Gy whole-body X-ray irradiation; 4.25 Gy separated by 3–6 h). Animals received isoflurane inhalant anesthesia prior to retro-orbital injections. Reconstitution was confirmed by flow cytometry detecting the ratio of CD45.1^+^ cells/CD45.2^+^ cells in the blood at 3 months post-transplantation (blood collected as described above). Low FSC/SSC population (i.e. lymphoid cells) and increased FSC/SSC population (i.e. granulocytes) were predominantly donor cells in all animals (low FSC/SSC median 90% donor, mean 89%, range 78–95%; increased FSC/SSC median 99% donor, mean 97%, range 82–100%) Reconstitution was similar in recipients of control and THS-exposed donor mice, as well as in recipients of male and female donor mice.

The recipient mice were followed for development of neoplasm or other illness for 1 year. Tissues including liver, spleen, lymph node, kidney, heart, lung and sternum of each animal were stored in 10% formalin, embedded in paraffin (sternum following decalcification), and the pathologic findings were assessed.

### Statistical analyses

Most statistical and survival analyses were performed using SPSS version 24, with statistical tests indicated in figure legends and in tables. Competing risk analysis was performed in R as described [21]. In regard to Supplementary Table S2: in order to decrease the risk of false positives as well as retain statistical power, we initially pre-selected 15 parameters for analysis by both nominal *P*-value and false discovery rate (FDR); these parameters are noted as ‘15parameters’ in EXCEL worksheet labels; further analyses performed in light of the initial statistical findings are noted as ‘added parameters’ in EXCEL worksheet labels.

## Results

### Experimental approach

**Figure 1:**
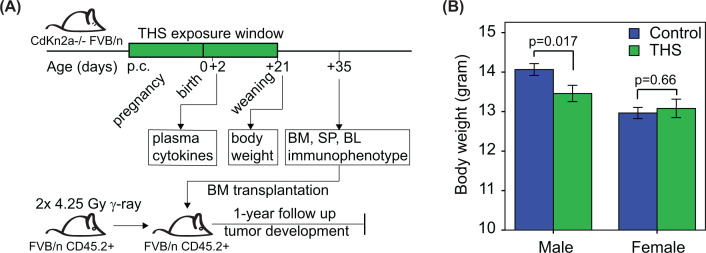
THS exposure altered body weight of 3-week-old male pups compared with control (**A**) Study design. Mice were exposed to THS starting from the first day of pregnancy (post coital, p.c.) until the pups were weaned at 3 weeks of age. Plasma cytokine levels were measured at 2 days of age. Body weight was assessed at weaning. BM, SP and PB were collected at 5 weeks of age for immunophenotyping and BM was transplanted into irradiated recipients. Tumor development was monitored for 1 year. (**B**) Bars represent body weight (gram) at weaning for control and THS-exposed male and female mice [*n*=142 pups (19 litters) in the Control group and 105 pups (15 litters) in THS-treated group]. Data are presented as the mean and error bars indicate standard error. *P*-values were obtained using the two-tailed *t* test.

To investigate the effects of *in utero* and early-life exposure to THS on the immune system and on leukemia/lymphoma risk, we exposed pregnant *Cdkn2a*^−/−^ dams to THS from the first day of pregnancy until weaning (Figure 1A). Plasma cytokine levels, body weight and hematologic parameters in BM, SP and PB were measured at different time points after birth. To determine the effect of THS exposure on leukemia/lymphoma risk, BM samples from THS-exposed and control *Cdkn2a*^−/−^ mice were transplanted into BM ablated (irradiated) wildtype recipient mice, which were then followed for 1 year.

### THS exposure significantly decreases body weight of male pups

We housed female *Cdkn2a*^−/−^ FVB/N mice with THS exposed cotton terrycloth swatches (5 × 5 cm^2^) from the first day of pregnancy until the pups were weaned at 3 weeks of age. Mice in the control group were housed with terrycloth swatches that were not exposed to THS. All cages also contained standard bedding material. The body weight of individual pups was measured on the day of weaning and included 142 pups (19 litters) in the Control group and 105 pups (15 litters) in THS exposed group. We observed a lower mean body weight of all pups in THS group (mean ± SEM: 13.27 ± 0.16 g) compared with control group (13.63 ± 0.12 g) and we found a statistically significant decrease in body weight of male pups in THS exposed group (13.44 ± 0.21 g; *n*=55) when compared with the male pups in the Control group (14.05 ± 0.15 g; *n*=87) (two-tailed *t* test, *P*=0.017) (Figure 1B). No difference in body weight was observed in female mice between the THS-treated group (13.07 ± 0.14 g; *n*=50) and the Control group (12.95 ± 0.23 g; *n*=55) (*t* test, *P*=0.66) (Figure 1B).

### THS exposure decreases cytokine levels in 2-day-old pups

**Figure 2:**
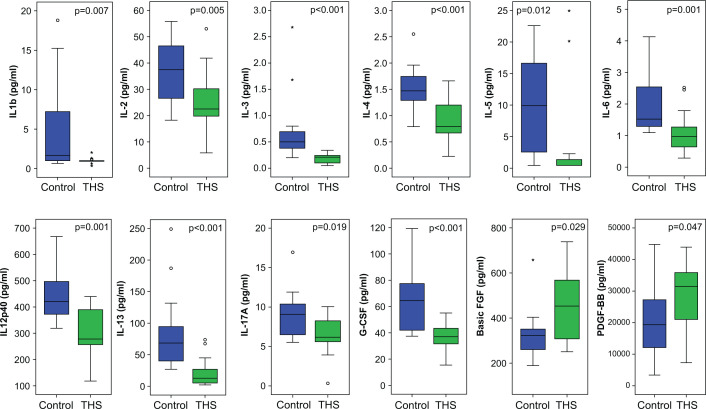
THS exposure affects plasma cytokine levels Boxplots of cytokine levels in 2-day-old mice exposed *in utero* to control (blue) or THS (green) (Control: *n*=16, one male and one female pup from 8 litters; THS: *n*=16; one male and one female pup from 8 litters). Box and whisker plots indicate median, 25^th^ and 75^th^ percentiles, 5^th^ and 95^th^ percentiles, and individual samples beyond these limits. Nominal *P*-values shown were obtained using the Mann–Whitney test. Outliers are cases with values beyond 1.5 times (°) and 3 times (*) the interquartile range. See also Supplementary Table S1 for FDR values.

To investigate the effect of THS exposure on plasma cytokine concentrations, we collected and isolated plasma from one male and one female pup at 2 days of age from each independent litter (*n*=16 for THS-exposed mice and *n*=16 for control mice) and measured concentrations of 32 cytokines (Supplementary Table S1; selected cytokines shown in Figure 2). We found that 20 out of 32 cytokines in THS exposed pups were lower than those in control mice including many interleukins (FDR < 0.1). Basic fibroblast growth factor (FGF) and the B-subunit of platelet-derived growth factor (PDGF-BB) were higher in THS exposed mice compared with control. Plasma cytokine differences were observed in both male and female mice (Supplementary Table S1).

### THS exposure affects the percentage of immune cell populations in BM, SP and blood

**Figure 3:**
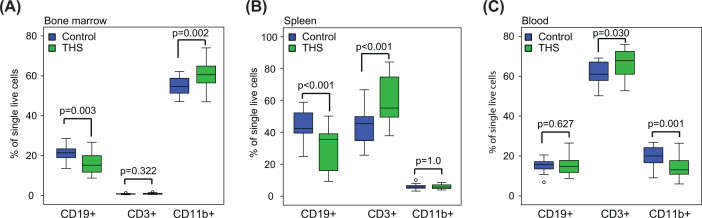
Comparison of immune cell populations by flow cytometry in different tissues of 5-week-old donor mice The percentage of B cells (CD19^+^), T cells (CD3^+^) and myeloid cells (CD19^−^/CD11b^+^) were measured by flow cytometry at 5 weeks of age in control (blue) and THS (green)-exposed mice (Control: *n*=19, one male and one female pup from 9 litters, one male from a 10^th^ litter; THS: *n*=20; one male and one female pup from 10 litters). (**A**) BM. (**B**) SP. (**C**) Blood. Box and whisker plots indicate median, 25^th^ and 75^th^ percentiles, 5^th^ and 95^th^ percentiles, and individual samples beyond these limits. Nominal *P*-values shown were obtained using the Mann–Whitney test. Outliers are cases with values beyond 1.5 times (°) the interquartile range. See also Supplementary Table S2 for FDR values.

To elucidate the potential influence of THS exposure on BM, splenic and blood cells we collected nucleated live cells of these tissues from one male and one female 5-week old mouse from independent litters and measured B cell, T cell and myeloid fractions by flow cytometry (Supplementary Table S2; Control: *n*=19, one male and one female pup from 9 litters, one male from a 10^th^ litter; THS: *n*=20; one male and one female pup from 10 litters). In BM, we observed a decreased percentage of B cells (FDR = 0.009) and an increased percentage of myeloid cells (FDR = 0.008) in THS exposed compared with control exposed mice (Figure 3A). In SP, we found a decreased percentage of B cells (FDR = 0.0005) and an increased percentage of T cells (FDR = 0.0005) in THS-exposed mice (Figure 3B). In blood, we found that THS exposed mice had an increase in the percentage of T cells (FDR = 0.045) and a lower percentage of myeloid cells (FDR = 0.0005) (Figure 3C).

**Figure 4:**
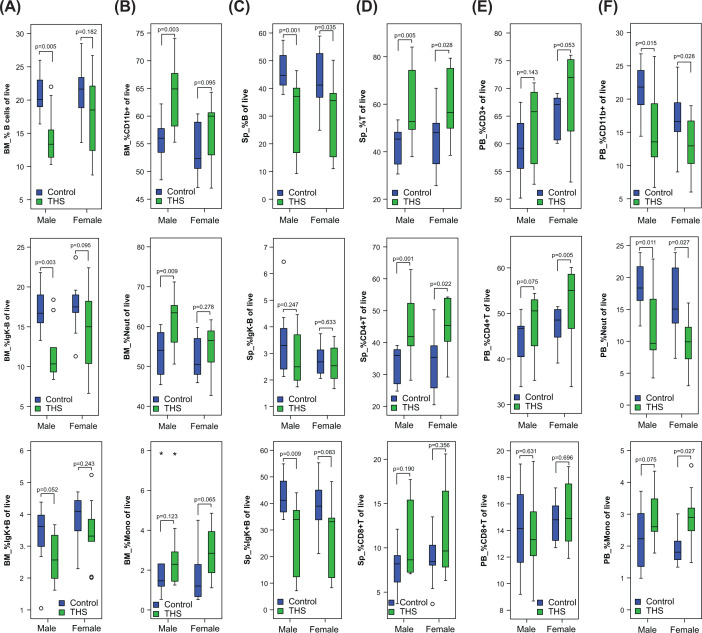
Sex-specific effects of THS on immune cell populations For immune cell populations identified in Figure 3 as divergent between control and THS-exposed mice, males and females, as well as immune subsets, were compared as described in the legend to Figure 3. (**A**) BM B cells (B220^+^/CD19^+^), mature B cells (B220^+^/CD19^+^/IgK^+^) and immature B cells (B220^+^/CD19^+^/IgK^−^). (**B**) BM myeloid cells (CD19^−^/CD11b^+^), monocytes (CD19^−^/CD11b^+^/Gr-1^neg-lo^) and neutrophils (CD19^−^/CD11b+/Gr-1^mod-hi^). (**C**) SP B cells (B220^+^/CD19^+^), mature B cells (B220^+^/CD19^+^/IgK^+^) and immature B cells (B220^+^/CD19^+^/IgK^−^). (**D**) SP T cells (CD3^+^), T-helper cells (CD3^+^/CD4^+^) and T-suppressor cells (CD3^+^/CD8^+^). (**E**) PB T cells (CD3^+^), T-helper cells (CD3^+^/CD4^+^) and T-suppressor cells (CD3^+^/CD8^+^). (**F**) PB myeloid cells (CD19^−^/CD11b^+^), monocytes (CD19^−^/CD11b^+^/Gr-1^neg-lo^) and neutrophils (CD19^−^/CD11b^+^/Gr-1^mod-hi^). Outliers are cases with values beyond 1.5 times (°) and 3 times (*) the interquartile range.

Given our observation that THS exposure particularly decreased the weight of 3-week-old male mice, the impact of sex was examined. In addition, analyses were performed to assess whether the observed differences in BM, spleen and blood were driven by changes in particular subpopulations (Supplementary Table S2). The decreased percentage of BM B cells and increased percentage of BM myeloid cells were more pronounced in male mice (Figure 4A,B). B-cell subpopulations in THS-exposed as compared with control exposed mice trended lower for both immature and mature B cells (nominal *P*-value <0.05 for immature marrow B cells in males), whereas myeloid subpopulations trended higher (nominal *P*-value <0.05 for marrow neutrophilic cells in males). In the spleen, the decreased percentage of B cells and the increased percentage of T cells were seen in both sexes, and were driven by decreased mature B cells and by increased CD4^+^ T cells (Figure 4C,D). The blood showed T-cell changes similar to but less pronounced than those seen in the spleen (Figure 4E, nominal *P*-value <0.05 for blood CD4^+^ cells in females), whereas in contrast with the increased percentage of BM neutrophilic cells seen in male mice, a decreased percentage of PB myeloid cells was seen in both males and females due to a decreased percentage of neutrophilic cells (Figure 4F).

### THS exposure alters the survival time of transplanted mice

**Figure 5:**
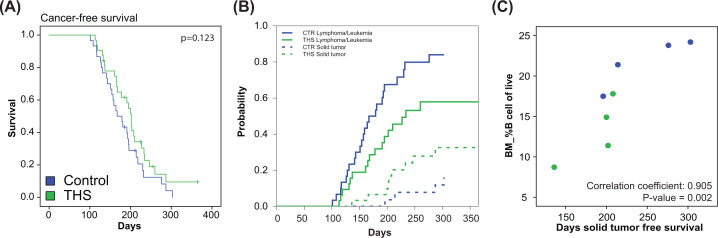
THS exposure significantly affects leukemia and lymphoma development (**A**) Cancer-free survival curves of BM recipient mice which received BM from control (blue; *n*=30) and THS (green; *n*=32)-exposed donor mice. *P*-value was obtained by Log-Rank Mantel–Cox test. (**B**) Cumulative incidence functions for competing risk of solid tumor development (dashed lines; *n*=5 for control, *n*=9 for THS) with leukemia/lymphoma (solid lines; *n*=24 for control, *n*=17 for THS) as first observed event (control mice indicated in blue and THS mice in green). Competing risk analysis: Control vs. THS *P*=0.02 for leukemia/lymphoma, *P*=0.13 for solid tumors. (**C**) Correlation between the percentage of transplanted B cells and tumor latency of non-leukemia/lymphoma cancers in the combined control and THS cohorts. Control mice indicated in blue and THS mice in green. *P*-value was obtained using Spearman correlation.

To investigate if THS promotes the development of hematopoietic tumors including leukemia/lymphoma, we transplanted BM isolated from 5-week-old control (*n*=30) and THS-exposed (*n*=32) mice (donor mice) to irradiated FVB/N congenic CD45.2 mice (recipient mice) (Figure 1A) (we hoped with this approach to reduce the risk that mice would become ill with non-leukemia malignancies that can also develop in *Cdkn2a^−/−^* mice.). Recipient mice were followed for tumor development for one year (Supplementary Table S3). We found that among the 30 recipient mice from control donor mice, 28 developed cancer within 1 year of transplantation. Among the 32 recipient mice from THS-exposed donor mice, 26 recipient mice developed cancer within 1 year. There was no significant difference in cancer-free survival between control and THS-exposed groups (Figure 5A; *P*=0.123). When focusing our analyses on the cause of death in the recipient animals, we observed trends towards later development of leukemia/lymphoma in THS-exposed animals and earlier development of solid tumors (Figure 5B; Control vs. THS *P*=0.02 for leukemia/lymphoma, *P*=0.13 for solid tumors). The significance of this observation was not entirely clear, and we considered the possibility that these differences in latencies reflected our particular experimental approach. One possibility was that THS immunosuppressive effects contributed to radiation-induced solid tumors in recipient animals. In our model, we used whole-body irradiation to ablate host BM prior to BM transplantation. BM-ablative radiation exposure significantly increases the risk of developing solid tumors (predominantly sarcomas). We therefore speculated that—if THS immunosuppressive effects accelerated the development of radiation-induced solid tumors in recipient animals—we would find that mice receiving lower numbers of B cells would have developed solid tumors at younger ages. There were 8 recipients of BM that developed such solid tumors and for which pre-transplant immunophenotyping data were available (4 = control exposed donors, 4 = THS-exposed donors). In these animals we indeed observed a significant correlation between the percentage of B cells in donor mouse marrow and days to solid tumor development (Figure 5C; Spearman rank correlation coefficient = 0.905; *P*=0.002); mice that received fewer B cells at transplant appeared to develop such non-leukemia/lymphoma cancers at earlier time points. Hence, the trends seen in Figure 5B could reflect THS immunosuppression of irradiated recipient animals leading to accelerated solid tumors, THS altering lymphopoiesis to delay leukemia/lymphoma, or a combination of these effects.

## Discussion

In the present study, we utilized the *Cdkn2a* null mouse model of childhood ALL to address *in utero* and early-life THS exposure effects, from the first day of pregnancy through weaning, on plasma cytokines, body weight, hematologic parameters and leukemia/lymphoma development. We found that THS exposure caused significant changes in plasma cytokine concentrations and in BM, SP and blood immune cell populations. We furthermore observed that THS exposure increased leukemia/lymphoma-free survival in BM transplantation recipient mice.

Since FVB/N mice that lack *Cdkn2a* are cancer-prone, primarily developing leukemia/lymphoma and sarcoma, we transplanted BM of THS-exposed and control *Cdkn2a* null mice into histocompatible BM-ablated recipient animals. As expected, we observed a high penetrance of leukemia/lymphoma in the recipient mice. Interestingly, in our model system recipient mice that received THS-exposed donor BM exhibited increased lymphoma/leukemia-free survival compared with recipient mice receiving BM from control donor mice. This result might reflect a consequence of the immunosuppressive effect observed in THS-exposed donor mice. The immunosuppressive effect results in fewer lymphoid cells from THS treated donor mice being transplanted into recipient mice compared with control donor mice effectively reducing the number of targets for oncogenic transformation and lowering the incidence of leukemia/lymphoma after THS exposure. However, an alternative explanation involving the risk of competing events has to be considered in our model since BM was transplanted after myeloablative radiation therapy of recipient mice. One side effect of this treatment is the development of solid tumors, which could prevent the observation of leukemia/lymphoma. It remains possible that the immunosuppressive effect of THS resulted in an acceleration in the development of these solid tumors, possibly due to inadequate immunosurveillance. Even though the development of solid tumors was not statistically different between THS-treated and control mice, we did observe an increased number of solid tumors that occurred earlier in the THS group. The inability to definitively conclude whether THS exposure influences solid tumor development is a limitation of our work. In conclusion, THS alone was not carcinogenic in our *Cdkn2a*^−/−^ leukemia/lymphoma model, but it may have contributed to radiation exposure-associated tumor development through its immunosuppressive effects.

Interestingly, epidemiological studies have suggested a relationship between smoking and a spectrum of diseases with a significant inflammatory component; in some cases there is evidence that smoking may decrease incidence and/or severity. For example, maternal smoking during pregnancy reduces the risk of type 1 diabetes in children [22]. Also, adult smoking reduced the risk of ulcerative colitis [23], sarcoidosis [24], endometriosis [25] and Parkinson’s disease [26]. A possible biological mechanism for these observations is that nicotine, which is present in cigarette smoke and THS, is known to have immunosuppressive effects [27]. Even though our results suggest that THS exposure might, in some settings, reduce the risk of leukemia/lymphoma, we observed profound potentially detrimental impacts on the immune system, the detrimental health risks associated with maternal smoking are well-documented, and any potential health benefit from the immunosuppressive effects of exposure to THS does not outweigh the harmful effects of smoking on health.

Future studies will have to determine whether the observed adverse effects of THS on hematologic parameters are dose- and genetic background-dependent. Our results show that THS exposure of FVB/n *Cdkn2a* null mice during pregnancy and early life has a profound effect on male body weight and on immune parameters in both males and females. In a related study, investigating effects of THS on body weight and hematologic parameters in C57BL/6 mice exposed during the first 3 weeks of life, we showed a reduction in body weight in both male and female mice [16]. Furthermore, our prior study showed that THS exposure during the first 3 weeks of life significantly increased the percentage of B cells in PB 14 weeks after THS exposure [16]. In contrast, our current study showed no difference in the percentage of B cells in PB and a significant decrease in SP and BM in THS-exposed mice at 5 weeks of age. These differences could be the result of differences in strain genetic background, exposure window and/or the time between exposure and immune parameter measurements. Chen et al., showed that THS exposure of C57BL/6 mice for 2 months starting at 3 weeks of age resulted in a dose-dependent increase in serum cytokine levels including IL-1a, IL-4, IL-10, TNFα and GM-CSF [15]. Similarly, Adhami et al., investigated exposure of male C57BL/6 mice to THS for 1, 2, 4 or 6 months starting at weaning and observed significant increases in serum levels of TNFα and GM-CSF when mice were exposed for as little as 1 month [14]. In our study, we also observed that THS exposure significantly altered plasma cytokine levels. However, in contrast with these previous studies showing a pro-inflammatory phenotype associated with THS exposure, we observed that perinatal exposure led to a significant decrease in the majority of cytokines assayed at 2 days of age, including IL-4, IL-10, TNFα and GM-CSF. The reason for these different observations could be due to differences in exposure window. Our cytokine measurements were conducted in mice exposed *in utero*, from the first day of pregnancy, to 2 days of age. THS exposure effects have not previously been investigated for this exposure. Development and maturation of the immune system starts early in fetal life and our results suggest that THS exposure affects this process. Differences could similarly be due to differences in mouse genetic background, experimental timing and/or exposure levels. Our study also found sex differences in the response to THS exposure emphasizing the importance of including both male and female mice in exposure studies. In general, male mice were more susceptible to THS exposure effects than female mice. Previous studies showed that male mice were found to be more sensitive than female mice to i*n utero* exposure to SHS for lung development and an immune challenge later in life [28,29]. These findings suggest that sex differences during fetal development play an important role in determining health risks associated with THS exposure. Collectively, these studies emphasize the need to define the window-of-susceptibility of THS-induced health outcomes. These studies can be initiated in mouse population-based model systems, which mimic the genetic and phenotypic diversity observed in the human population while allowing precise control of exposures and the ability to analyze multiple phenotypic endpoints [30].

In conclusion, our results using the *Cdkn2a*^−/−^ mouse model of leukemia/lymphoma showed that THS exposure during pregnancy and early life caused substantial biological effects, including decreased regulators of the immune system at birth (cytokines) and persistent alterations of blood cells. These findings further support the growing evidence that THS exposure may have significant persistent health effects for human mothers and infants. Although our results did not demonstrate that THS exposure increased risk for leukemia/lymphoma, its immunosuppressive effects may have contributed to the carcinogenic effects of ionizing radiation. These data contribute to our understanding of the potential health risks of THS exposures, and should be useful for framing and advocating for policies against indoor smoking in the U.S.A. and worldwide.

## Clinical perspectives

- We investigated the effects of *in utero* and early-life THS exposure on plasma cytokines, body weight, hematologic parameters and leukemia/lymphoma development using the *Cdkn2a* null mouse model of childhood ALL.
- Our study demonstrates that *in utero* and early-life THS exposure is broadly immunosuppressive and increased leukemia/lymphoma-free survival in BM transplantation recipient mice.
- Our study adds to expanding evidence that THS exposure has profound effects on the immune system and that *in utero* and early life developmental periods represent vulnerable windows of susceptibility for these effects.

## Supplementary Material

Supplementary Tables S1-S4

## Data Availability

Datasets related to the article are included as Supplementary Tables S1–S4.

## Abbreviations

ALL: acute lymphoblastic leukemia
BM: bone marrow
FI: fluorescence intensity
FSC: forward scatter
PAH: polycyclic aromatic hydrocarbons
PB: peripheral blood
SHS: secondhand smoke
SP: spleen
SSC: side scatter
THS: thirdhand smoke
